# Management of Chronic Pain in Elderly Patients: The Central Role of Nurses in Multidisciplinary Care

**DOI:** 10.3390/geriatrics10040110

**Published:** 2025-08-14

**Authors:** Dorina Markovics, Andrea Virág, Klara Gadó

**Affiliations:** 1 Department of Geriatrics, and Centre of Nursing Sciences, Semmelweis University, 1097 Budapest, Hungary; 2 Department of Clinical Studies, Faculty of Health Sciences, Semmelweis University, 1088 Budapest, Hungary

**Keywords:** chronic pain, elderly, geriatric, painkillers, analgesic, nonsteroidal anti-inflammatory drugs, opioids

## Abstract

Pain is a fundamental yet complex biological and psychosocial phenomenon. While acute pain serves as a defense mechanism, alerting the body to potential tissue damage, chronic pain loses this protective function and becomes a persistent, independent condition. Chronic pain in the elderly is particularly significant due to age-related changes in pain perception, a higher prevalence of comorbidities, and an increased susceptibility to pharmacological side effects. Diagnosing pain in older adults presents unique challenges owing to cognitive decline, multimorbidity, and impaired communication. This narrative review aims to summarize the current knowledge on chronic pain in the elderly, with a particular emphasis on diagnostic difficulties, therapeutic strategies, and the essential role of nurses in multidisciplinary management. Both objective scales and subjective assessment tools are essential for an accurate evaluation. Effective management requires a multidisciplinary approach that integrates individualized pharmacological and non-pharmacological therapies. Analgesic use must be tailored to account for altered pharmacokinetics and risks such as sedation or falls. Non-drug interventions, including physiotherapy and psychological techniques, are especially valuable in geriatric care. Nurses play a pivotal role in the recognition, assessment, and ongoing management of pain in this population. Developing age-appropriate, personalized strategies is essential for improving the quality of life in older adults living with chronic pain.

## 1. Introduction

As the proportion of aging populations increases, greater attention is being paid to the health issues associated with aging [1]. Among these, chronic pain is particularly significant due to its profound impact on the physical, mental, and social aspects of life. Persistent pain in the elderly can lead to cognitive decline, exacerbate social isolation, and significantly reduce quality of life [2]. Effective pain management is crucial not only to alleviate individual suffering but also to maintain the patients’ independence, social connections, and overall well-being [3]. Given the complex etiology of chronic pain, its management often requires a multidisciplinary approach, encompassing pharmacological therapy, physiotherapy, psychotherapy, and lifestyle modifications [4]. This paper examines the unique characteristics of pain in older adults, as well as the diagnostic and therapeutic options tailored to this age group and the relevant geriatric pain management guidelines, with a focus on the risks and benefits of different treatment modalities in the elderly.

### Identifying Research Gaps in Current Literature

Although numerous reviews have addressed the prevalence, pathophysiology, and management of chronic pain in older adults, important gaps remain in the current evidence base. First, most available studies are cross-sectional in nature, providing limited insight into the longitudinal trajectories of chronic pain and its impact on the functional, cognitive, and psychological outcomes in the elderly. Second, despite widespread reference to the biopsychosocial model, few reviews have systematically examined how the biological, psychological, and social determinants interact over time in shaping the pain experience and the treatment response in older populations.

Third, while non-pharmacological interventions—such as physiotherapy, cognitive behavioral therapy, and complementary therapies—are frequently recommended, the data on their efficacy, accessibility, and integration into routine care for older adults remain limited. Fourth, the role of nurses in chronic pain management is often acknowledged but insufficiently explored in the literature, particularly regarding their practical contributions, the perceived barriers, and their training needs. Finally, previous reviews rarely incorporate evidence and gap maps that systematically identify the areas of strong evidence versus the under-researched domains, especially in populations with multimorbidity, frailty, or cognitive impairment.

This review aims to address these gaps by providing a comprehensive synthesis of the current knowledge on chronic pain in elderly patients, with a particular emphasis on the central role of nurses, multidisciplinary approaches, and the practical challenges to effective assessment and management.

To support this narrative review and to address these gaps, we searched the PubMed database for articles published in the last five years using keywords such as ‘chronic pain’, ‘elderly’, ‘geriatric’, ‘painkillers’, ‘analgesic therapy’, ‘nonsteroidal anti-inflammatory drugs’, ‘opioids’, ‘non-pharmacological pain relief’, and ‘role of nurses’. Priority was given to original research articles and systematic reviews relevant to geriatric populations.

## 2. Types of Pain

Pain can be categorized into acute and chronic pain. Acute pain is a short-term response to injury, tissue damage, or disease. It typically begins suddenly and lasts for a few days to weeks. Acute pain is often associated with a clear cause, such as a wound, fracture, infection, or surgery. Its primary function is to act as a warning signal, prompting protective behaviors to avoid further harm [5]. In contrast, chronic pain persists for more than three months and often lacks a clear physiological cause. Chronic pain is frequently multifactorial and can have long-term detrimental effects on a patient’s quality of life [6]. Common types of chronic pain include the following: Osteoarthritis pain; caused by joint degeneration and inflammation, highly prevalent in older adults. Neuropathic pain: arises from nerve damage or injury, often characterized by burning, numbness, or stabbing sensations. Cancer-related pain; frequently intense and challenging to manage. Fibromyalgia: manifests as widespread muscle and connective tissue pain, often accompanied by fatigue and sleep disturbances. Chronic low back pain results from spinal degeneration or other causes, leading to significant mobility limitations. Visceral pain: stems from chronic pain in internal organs such as the abdomen or pelvis and is often difficult to diagnose [1].

## 3. Why Focus on Older Adults?

This review focuses on older adults as a population disproportionately affected by chronic pain. Chronic pain is significantly more prevalent among older adults compared to younger individuals. Additionally, the perception, experience, and response to pain and its consequences vary distinctly in this population. Chronic pain profoundly influences the social circumstances, quality of life, psychological well-being, and cognitive functions of older adults.

## 4. Prevalence of Chronic Pain in Older Adults

Chronic pain is more prevalent among older adults compared to younger populations. In the US, it was reported that 30.8% of adults aged over 65 experienced chronic pain, compared with 8.5% of those aged 18–29 and 14.6% of those aged 30–44 [7].

For the population living in nursing homes, this rate can reach 80% [8]. National and European surveys underscore chronic pain as a major health issue in the elderly, with rates potentially three times higher than in the younger populations. The risk of chronic pain increases with age and comorbidities, such as cancer, diabetes, and peripheral neuropathy [9,10,11]. Additionally, diminished pain perception in conditions like coronary artery disease can lead to diagnostic challenges. These findings underscore the significance of effective pain management for individual well-being and for the overall functioning of healthcare systems.

## 5. Psychological Changes and Cognitive Decline

### 5.1. The Role of Pain and Psychological Stressors in Accelerating Cognitive Decline

Conditions such as depression and anxiety, which are more common in older adults, can amplify the subjective experience of pain. Social isolation, loneliness, and a lack of environmental stimulation can further exacerbate the perception of pain. Chronic pain in older adults is closely linked to cognitive decline. Studies indicate that persistent pain can impair memory, attention, and executive functions. Chronic pain can lead to structural and functional changes in the brain, including a reduced hippocampal volume and the overactivation of pain-processing areas such as the anterior cingulate and the prefrontal cortices. These changes contribute to cognitive overload and decreased resilience [12,13]. Sleep disturbances, often associated with chronic pain, exacerbate cognitive decline by impairing memory consolidation and concentration [14]. Psychosocial factors, including reduced activity levels and social isolation, also accelerate cognitive decline. Multidisciplinary pain management approaches that incorporate psychological support, physical activity, and improved sleep quality are crucial in mitigating these effects.

### 5.2. Pain in Patients with Dementia

Dementia significantly influences both the prevalence and recognition of pain in older adults. While individuals with dementia experience pain at rates comparable to their cognitively intact peers, their ability to report it is often diminished due to communication deficits, memory loss, or impaired insight. This underreporting can lead to undertreatment and poorer outcomes. Furthermore, neurodegenerative changes in the brain regions involved in pain processing, such as the prefrontal cortex and the limbic system, may alter pain perception and behavioral responses. Studies suggest that in advanced dementia, pain may manifest through nonverbal cues such as facial expressions, vocalizations, agitation, or changes in appetite and sleep. Therefore, pain prevalence may be underestimated unless appropriate observational tools like the PAINAD (Pain Assessment in Advanced Dementia) scale are used [2,15,16].

### 5.3. Undertreatment of Pain in Older Adults

Undertreatment of pain is a critical and well-documented problem in geriatric care. Older adults frequently receive inadequate pain relief due to various factors, including the underreporting of symptoms, the misattribution of pain to normal aging, and the reluctance of clinicians to prescribe potent analgesics. Concerns about side effects, polypharmacy, and the risk of addiction—especially with opioids—often result in overly cautious prescribing practices. This therapeutic hesitation may lead to prolonged suffering, reduced functional ability, and a diminished quality of life. Addressing undertreatment requires greater awareness among healthcare professionals, the use of appropriate assessment tools, and individualized treatment strategies that balance efficacy with safety.

## 6. Pain Perception

Pain perception is a complex process initiated by nociceptors, the sensory receptors for pain. These receptors are the endings of the sensory nerves, responding to various stimuli such as temperature changes, pressure, or chemical signals. Once detected by nociceptors, the pain signals are transmitted through different levels of the nervous system—peripheral nerves, the spinal cord, and the brain. The brain is where both the sensation and the emotional response to pain are processed, involving key centers like the thalamus and the limbic system.

In older adults, pain perception and processing undergo several changes. Age-related alterations in the central nervous system, such as a reduction in the number of neurons and slower neural conduction, can influence pain sensitivity. Moreover, the responses to pain may differ due to changes in the neural pathways and neurotransmitter function. As a result, older individuals may experience heightened or diminished pain sensations, with differences in how the pain is processed compared to the younger populations [17].

## 7. Changes in Pain Perception with Aging

Physiological changes associated with aging significantly affect the perception and management of chronic pain in older adults. For instance, the peripheral nervous system experiences a decline in the number and function of nociceptors, potentially altering pain sensitivity. Consequently, older individuals may exhibit reduced or atypical responses to certain pain stimuli, complicating pain recognition and treatment. Research has consistently shown a decreased sensitivity to lower pain intensities in aging populations [17].

The central nervous system also undergoes significant age-related changes, such as a reduction in the neuronal density, disruptions in the neurotransmitter balance, and functional decline in the brain structures like the prefrontal cortex and the hippocampus, which are critical for pain processing. These changes can contribute to both heightened pain sensitivity and diminished pain responses.

Psychosocial factors play a crucial role in pain perception among older adults. Cognitive decline, particularly related to memory and attention, can hinder accurate pain recognition and communication, complicating medical interventions [18]. Additionally, depression and anxiety, common in this age group, can amplify the subjective experience of pain and its intensity.

## 8. Pain Assessment in Older Adults

Assessing pain in older adults is particularly challenging due to its subjective nature, influenced by individual perception, prior experiences, and the psychological state [19]. Pain is a subjective experience shaped by multiple factors, including age, psychological status, and prior encounters with pain. Since there is no objective tool that can precisely measure pain intensity, clinicians must rely entirely on the patients’ subjective accounts. This approach underscores the principle that the patient’s report is always valid; if someone reports that they are experiencing pain, it is a genuine experience for them, even if the environmental or medical evaluations do not clearly corroborate it.

Recognizing the subjective nature of pain is crucial for its management. The patient’s feelings and descriptions must be considered to design an individualized treatment plan. Pain is not merely a reflection of a physical condition but also involves a psychosocial dimension that significantly influences its perception and impact. Effective management entails addressing this multidimensional nature of pain, ensuring that both its physical and psychosocial aspects are considered.

Pain assessment is a multifaceted process that requires various methods due to the subjective nature of pain [20]. Self-reporting remains the most common approach, where patients describe their pain verbally or through visual tools. This method is highly effective as it directly relies on the patient’s experience, providing invaluable information due to the subjective nature of pain. Verbal scales, such as the 0–10 numeric rating scale, are simple and quick to administer [21]. However, the accuracy of self-reporting may decrease in older patients, particularly in those with cognitive impairments [22]. A visual analogue scale (VAS), where patients indicate their pain level on a continuum, offers an alternative. These are easily understood and efficient, particularly for assessing acute pain and tracking its changes over time. Nevertheless, they may pose challenges for individuals with motor or visual deficits, limiting their applicability in elderly or physically debilitated patients.

Observational scales are especially useful in cases where patients cannot communicate. For instance, the Pain Assessment in Advanced Dementia (PAINAD) scale evaluates pain based on observable indicators such as facial expressions, vocalizations, body posture, or activity levels. Although these methods are inherently subjective, they play a critical role for caregivers and family members in identifying pain in patients with dementia or severe cognitive decline [22]. Observations made by nurses or family members are vital for classifying pain-related behaviors and tailoring the appropriate interventions.

Various studies have explored the effectiveness of different pain assessment methods. Physiological markers, such as changes in the heart rate, blood pressure, or cortisol levels, are well-documented responses to pain, though other stress factors can influence these indicators. Emerging technologies, such as electroencephalography (EEG) and functional imaging techniques like functional magnetic resonance imaging (fMRI), offer new possibilities for pain measurement. However, their high cost and limited accessibility currently restrict their use in research settings [23].

Psychometric tools, such as the McGill Pain Questionnaire [24], enable the measurement of both the qualitative and quantitative aspects of pain. Despite their utility, these tools can be time-consuming and are less practical for older or physically frail patients. Combining objective and subjective data, such as physiological markers and self-reporting methods, provides the most effective approach to understanding the complexity of pain [25].

**Figure 1:**
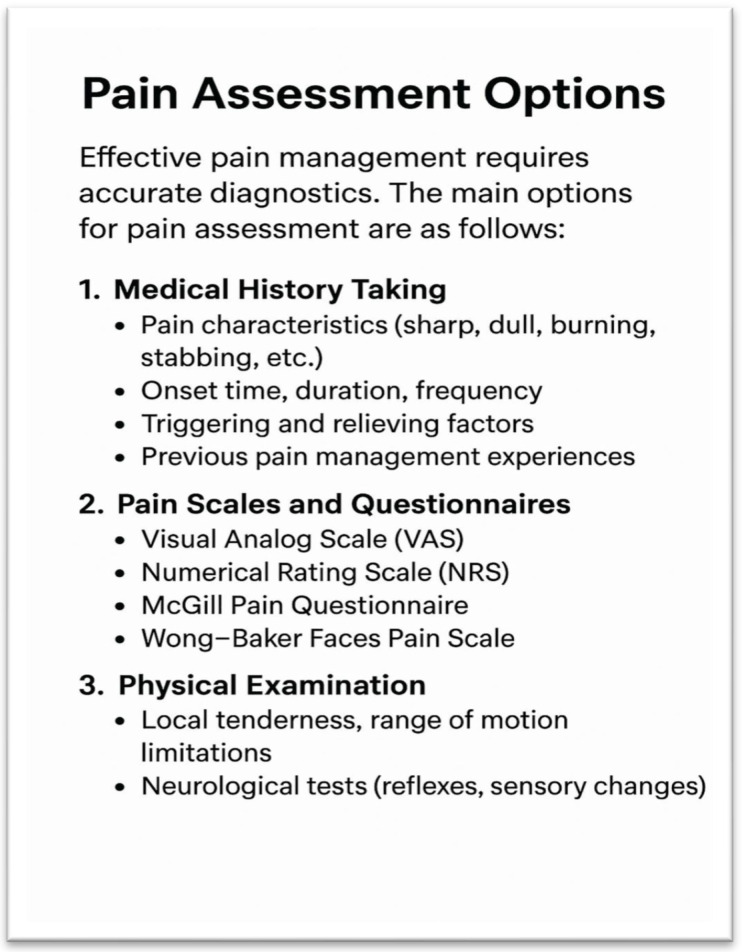
Overview of pain assessment methods (structured checklist) (own illustration).

**Figure 2:**
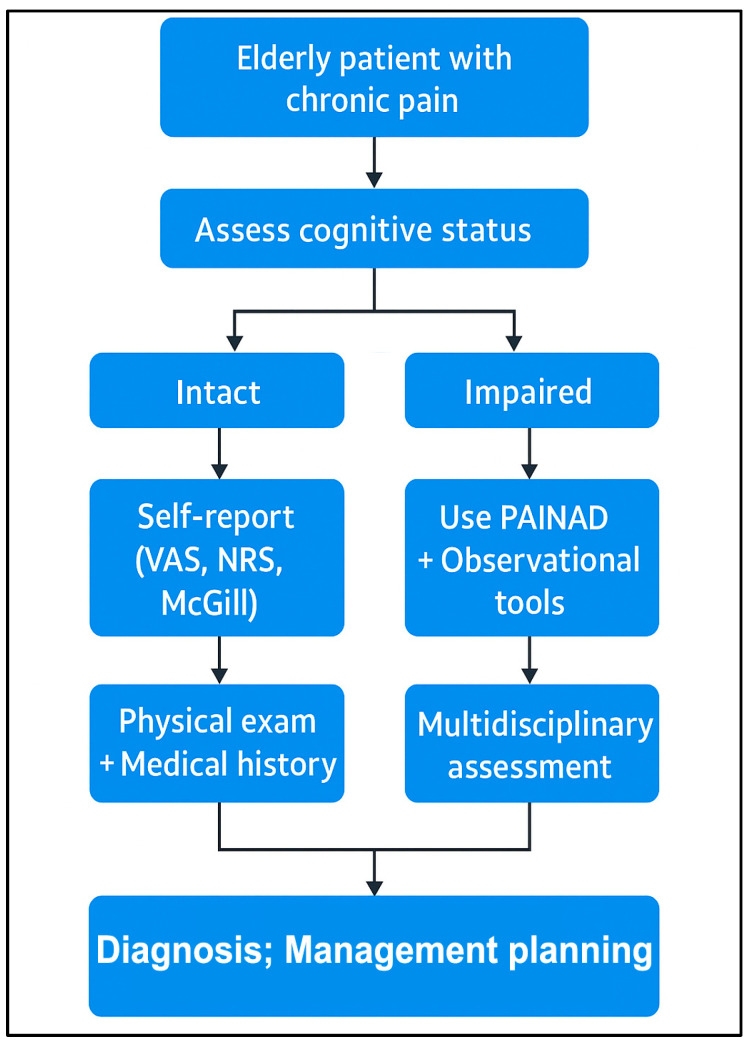
Cognitive status-adapted flowchart for chronic pain evaluation in elderly patients (own illustration).

The objectification of pain also requires attention to the physical parameters that complement the subjective reports. Changes in the blood pressure, heart rate, respiratory rate, and skin coloration can signal the presence of pain. Additionally, involuntary reactions, such as changes in facial expressions, muscle tension, or altered posture, provide valuable insights into the pain intensity. For example, if a patient protects a painful area or avoids movement, this behavior may indicate an underlying condition. Pain assessment thus relies not only on verbal complaints but also on nonverbal cues, contributing to a more accurate evaluation and the development of appropriate treatments [26,27,28] (Figure 1 and Figure 2).

## 9. Age-Specific Challenges in Pain Management for Older Adults

Pharmacological pain management presents unique challenges in older adults, who are more susceptible to the side effects of analgesics such as opioids or non-steroidal anti-inflammatory drugs (NSAIDs). While opioids can cause sedation, confusion, and constipation, NSAIDs can worsen renal function, cause hypertension, and provoke gastric ulcers.

Non-pharmacological strategies, including physiotherapy, cognitive behavioral therapy, and lifestyle modifications (e.g., increased physical activity), can also be effective. These approaches not only reduce the pain but also enhance the overall quality of life for older patients. Effective pain management for older adults requires a comprehensive approach that addresses the biological, psychological, and social factors [19].

## 10. Role of Nurses in Chronic Pain Management in the Elderly

Nurses play a pivotal role in the care of elderly patients, particularly when addressing chronic pain. Relieving pain in older adults is not only essential for improving their quality of life but is also a fundamental requirement for ensuring the effective and safe delivery of nursing care. This section explores why nurses hold such a central role in pain management and the key considerations they must take into account [25].

Nurses are the healthcare professionals most closely connected to patients. In the daily and long-term care of older adults, their continuous presence allows them to thoroughly understand the patient’s condition, complaints, and other needs. Older adults often experience a combination of physical and psychological symptoms that can only be adequately addressed when the nurse has detailed information about their state.

Nurses can identify the signs of chronic pain, even when the patient cannot or will not articulate them. Older adults often tend to downplay their complaints, believing that pain is an inevitable part of aging. Through empathetic approaches and experience, nurses can help bring these issues to light.

Chronic pain affects not only the patient but also the nurse’s workload. Pain often makes patients less able or willing to cooperate with nursing tasks such as bathing, feeding, or mobilization. This not only complicates the nurse’s work but also increases the risk of complications such as pressure ulcers. For example, pain during movement may hinder a patient’s rehabilitation. In such cases, nurses must not only manage the pain but also devise alternative strategies to mobilize the patient while minimizing discomfort. Untreated pain frequently leads to psychological stress, anxiety, or depression, further distancing the patient from an improved quality of life.

Nurses play a crucial role in collecting and communicating information about the patient. They act as the eyes and ears of the medical team, continuously monitoring the patient’s condition and reporting any changes. This is particularly important for older adults, as patients with multiple chronic illnesses can experience rapid and subtle deteriorations. Thanks to their constant presence, nurses can accurately detect changes in the patient’s pain levels and relay this information to the physician. This is especially significant because medical visits do not always provide sufficient opportunities to gather comprehensive pain-related information from the patient.

The role of the nurse extends far beyond physical care. Chronic pain often brings feelings of loneliness and social isolation. Nurses, through empathy, attention, and support, can help patients emotionally cope with their situation. Emotional support is often as critical as the pain management therapy itself.

Nurses are vital members of the multidisciplinary team working to alleviate pain in older adults. Collaboration among physicians, physical therapists, dietitians, and nurses is essential to provide comprehensive and holistic care. Within this team, the nurse ensures that the medical instructions are effectively implemented in practice.

**Figure 3:**
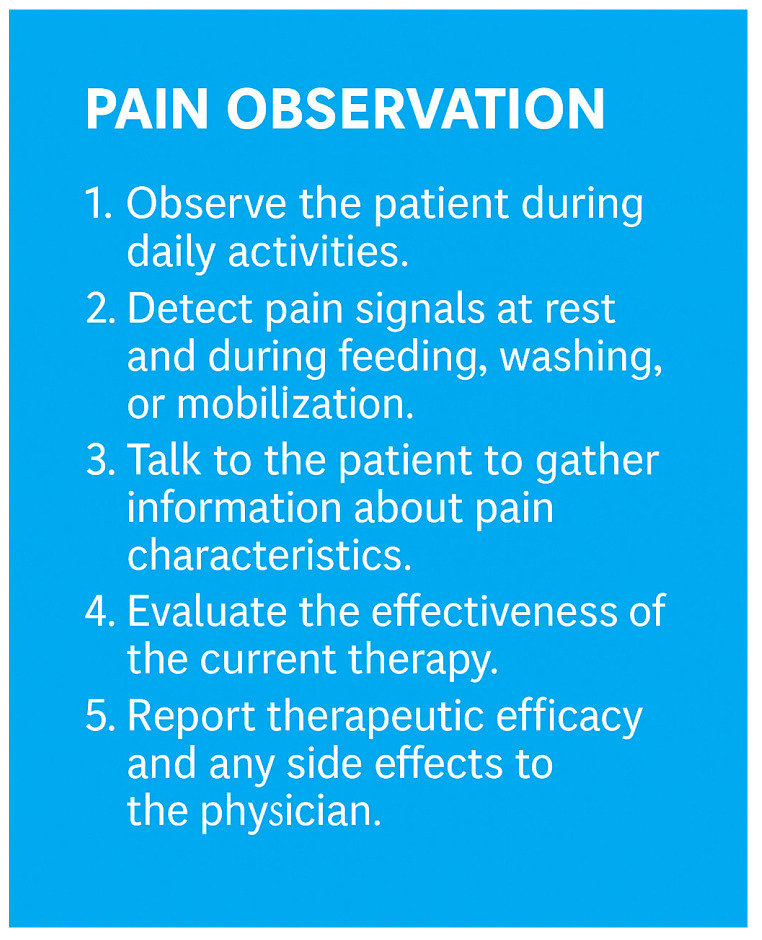
Key steps in the nurse’s role in chronic pain management in elderly patients (own illustration).

Nurses are also skilled in documenting the pain intensity, location, and characteristics in detail, which is fundamental for selecting the appropriate therapy. Their observations form the basis of a tailored and effective pain management plan (Figure 3).

## 11. Pain Relief Options: A Discussion of Drug and Non-Pharmacological Therapy

Pain is one of the most fundamental and complex biological and psychological phenomena in human life [29]. While acute pain is a physiologically useful defense mechanism that alerts the body to potential or existing tissue damage, chronic pain is not [30]. Persistent pain not only causes physical suffering but can also significantly impair quality of life, cause psychological distress, and limit daily activities. This can lead to social isolation and depression [13].

A plethora of therapeutic options exist to alleviate this condition, which can be broadly categorized into two primary classifications: drug-based interventions and non- pharmacological treatments [31]. The selection of an appropriate treatment is contingent on a multitude of factors, including the etiology, intensity, and duration of the affliction [29]. Acute pain, by virtue of its transitory nature, is typically addressed through the implementation of targeted, short-term therapeutic interventions. Conversely, the management of chronic pain, which is characterized by its multifactorial origins and complex pathophysiology, necessitates an integrated, multidisciplinary approach that encompasses diverse therapeutic modalities.

Effective management of pain frequently necessitates a multifaceted approach, encompassing various therapeutic modalities, including pharmacological treatments, physiotherapy, psychological interventions, and lifestyle modifications. This holistic strategy is designed not solely to alleviate pain but also to enhance the patient’s quality of life, restore their functional capabilities, and optimize their long-term health outcomes.

## 12. Pharmacological Management of Pain

Medication represents one of the predominant methods of providing rapid and effective pain relief. Medicines exert their effects via diverse mechanisms, thus enabling the treatment of a variety of pain types. Analgesic drugs can be categorized into three primary categories: minor and major analgesics, and adjuvant drugs.

### 12.1. Minor Analgesics

These are employed in the management of pain of mild to moderate intensity. Representing a highly prevalent class of analgesics, non-steroidal anti-inflammatory drugs (NSAIDs) that are frequently prescribed for the management of pain and inflammation include ibuprofen and diclofenac. Beyond their ability to alleviate discomfort, NSAIDs also demonstrate substantial anti-inflammatory properties, making them particularly efficacious in the treatment of pain associated with inflammation [32].

NSAIDs, such as ibuprofen, diclofenac, and naproxen, provide moderate to strong analgesic and anti-inflammatory effects but may cause gastrointestinal irritation, bleeding, renal impairment, and pose an increased cardiovascular risk. Metamizole offers strong analgesic and antipyretic effects with fewer GI side effects, though it carries a rare risk of agranulocytosis. Paracetamol (acetaminophen) has mild to moderate analgesic and antipyretic effects, minimal GI or cardiovascular risks, but may cause hepatotoxicity at high doses [33].

Paracetamol, another widely used analgesic, is particularly effective against mild pain and has a low potential for adverse effects. However, it is important to note that at high doses, it can cause liver damage, so it is essential to adhere to the recommended dosage [34] (Table 1).

Paracetamol has the fewest side effects, while diclofenac and metamizole have stronger effects but higher risk profiles.

### 12.2. Major Analgesics

Major analgesics are used for managing severe pain, whether from malignancies or non-malignant conditions. These include opioids such as morphine, fentanyl, tramadol, and oxycodone. Opioids are highly effective in managing severe pain, including pain from surgery, cancer, osteoarthritis, vertebral fractures, and advanced degenerative joint disease [35].

Tramadol is often preferred for moderate pain, due to its lower risk of respiratory depression compared to the stronger opioids. However, tramadol may increase the risk of confusion and fall in frail elderly patients, which requires careful monitoring [36]. Tramadol has been demonstrated to reduce the seizure threshold, thereby increasing the probability of seizures. This, in turn, results in an elevated risk of falling. Consequently, the utilization of tramadol should be initiated at the lowest effective dose for elderly patients [37].

Morphine remains a standard for severe pain, especially for cancer pain, but its use in older adults requires careful dose adjustments due to its renal metabolism. Reduced renal function in older patients can slow morphine clearance, increasing the risk of accumulation and toxicity.

Oxycodone is another opioid option for severe pain, and like morphine it may need to be adjusted to account for renal function. It has a somewhat more predictable pharmacokinetic profile than morphine, which can make it a good alternative for patients with impaired renal function.

Transdermal fentanyl is suitable for patients with stable chronic pain, especially those who cannot tolerate oral medications. This long-acting formulation provides consistent pain relief, making it a good option for elderly patients with stable pain management needs.

Opioids should not be reserved solely for cancer-related pain. They also play an important role in managing severe non-malignant pain, such as osteoarthritis, complex fractures, and degenerative joint disease. The use of opioids in these cases must be carefully weighed against the risk of side effects such as sedation, confusion, and gastrointestinal problems [38].

Combination therapy, for example, an opioid combined with paracetamol or a non-steroidal anti-inflammatory drug (NSAID), may be used to lower the opioid dosage and minimize the side effects while maintaining pain relief efficacy [39,40].

The correct use of opioids is complicated by a fear of addiction and tolerance. When opioids are used to treat cancer pain, the development of dependence should not be a major concern [41]. The use of opioids in such cases is not only to reduce pain, but also to ensure a dignified life for the patient. For patients with terminal illnesses, pain relief is an eminent part of maintaining their quality of life. Therefore, the use of opioids in these patients is necessary and justified rather than dangerous [42]. They are used under medical supervision, under controlled conditions, and the dosage can be adapted to account for the patient’s pain level. Medical necessity and the patient’s quality of life are paramount, and the risk of addiction is minimal if treatment is administered appropriately (Table 2).

### 12.3. Adjuvant Medications

Adjuvant medications are not primarily indicated for pain relief but are very effective in treating certain types of pain or complementing other medications. Examples include antidepressants (e.g., tricyclics or SSRIs), which can be used to treat chronic pain such as neuropathic pain [43]. Similarly, anticonvulsants (e.g., gabapentin, pregabalin, carbamazepine) are also used to relieve neuropathic pain [44]. The aim of these drugs is not only to reduce pain, but also to affect the neurological processing of the pain sensations.

### 12.4. Age-Specific Considerations in Pharmacological Pain Management

Older adults present unique challenges in pharmacological pain management due to altered pharmacokinetics and pharmacodynamics. Aging-related renal and hepatic impairments slow drug metabolism and clearance, increasing the risk of drug accumulation and toxicity. This is especially critical when prescribing opioids, as older adults are more susceptible to the adverse effects of opioids, which include sedation, confusion, and respiratory depression.

Due to the reduced renal function in elderly patients, opioids such as morphine and tramadol may accumulate, leading to increased side effects and toxicity. Therefore, careful dose adjustments and close monitoring are essential [45]. Polypharmacy, which is common in geriatric populations, can also complicate therapy due to potential drug–drug interactions that may exacerbate the side effects or diminish the effectiveness of the pain management [46].

Moreover, older adults are more vulnerable to side effects like sedation, confusion, falls, and gastrointestinal bleeding. The increased risk of opioid-induced delirium and falls in frail elderly patients is a particular concern. Even low doses of opioids may cause confusion or postural instability, increasing the risk of falls and fractures, which can lead to serious complications, including hospitalization and death [47]. Opioid-induced respiratory depression is also a significant risk, particularly in older adults with already compromised respiratory function. The risk can be reduced by correctly applying the “starting low and going slow” rule.

Additionally, constipation (obstipation) is a common issue in older patients and can be worsened by opioids, further complicating their pain management. Careful monitoring and supportive measures such as laxatives or stool softeners may be necessary to manage this side effect.

When selecting an opioid for older patients, individual factors such as cognitive status, prior history of falls, and frailty must be considered. Extended-release formulations of opioids may be appropriate for patients with stable chronic pain, while short-acting formulations are better suited for breakthrough pain. Combining opioids with non-opioid analgesics (e.g., paracetamol, NSAIDs) can help to reduce the required opioid dose and minimize the risk of side effects while maintaining effective pain relief.

Frequent reassessments of pain and medication efficacy are crucial in elderly patients, and dose adjustments to optimize both safety and comfort are essential [47].

## 13. Medication Strategy for Pain Relief

In the case of medication for pain relief, it is important not only to determine the type and dosage of the medication, but also to adjust the route and frequency of administration.

**Figure 4:**
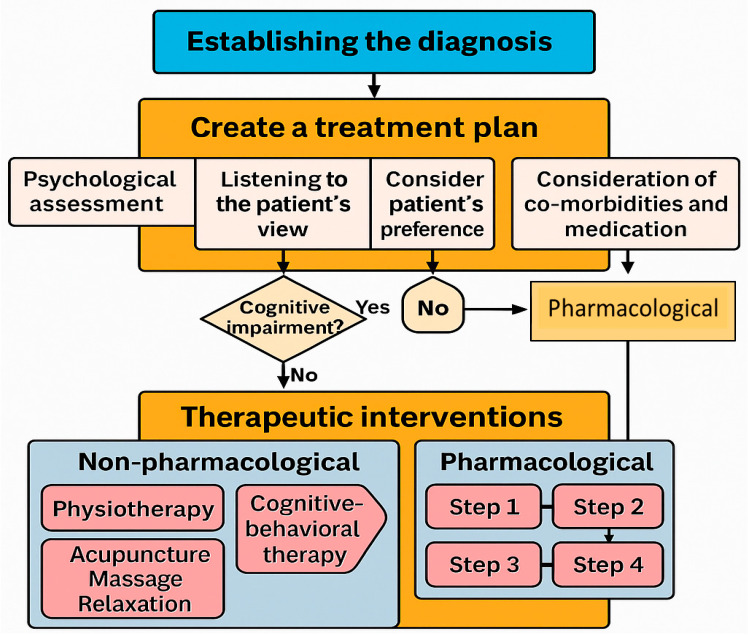
Decision-making flowchart for pharmacological and non-pharmacological pain interventions (own illustration).

The basic principles of pharmacological analgesia include a stepwise therapeutic approach using different analgesics depending on the intensity of the pain [48] (Figure 4).

**Figure 5:**
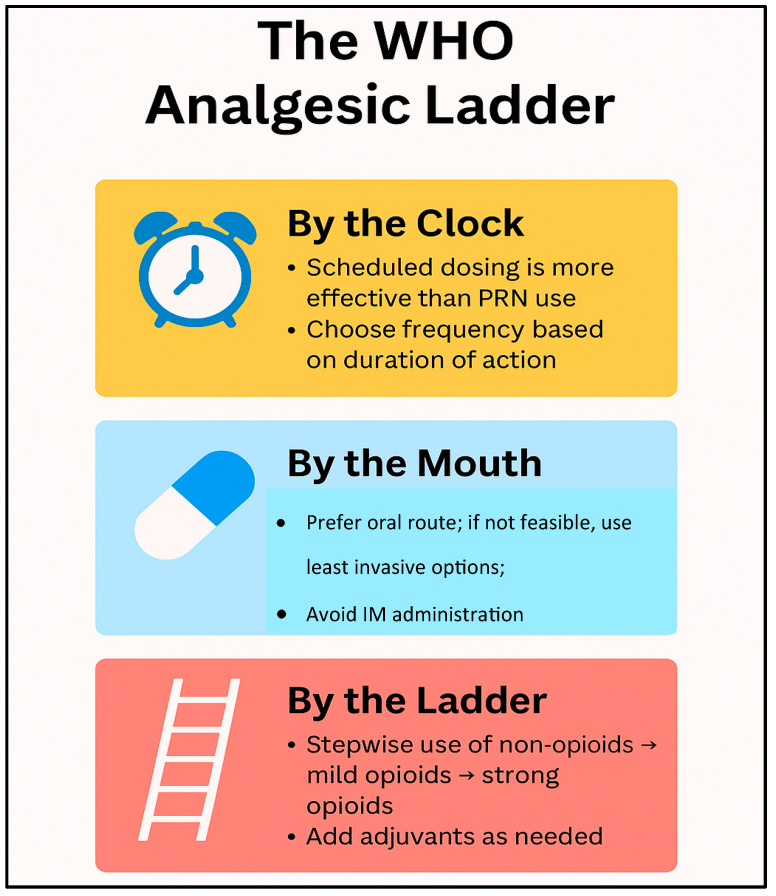
Key findings associated with the WHO analgesic ladder [49].

There are three main levels of stepwise analgesia (the WHO pain ladder): level one for mild pain, level two for moderate pain, and level three for severe pain [46] (Figure 5). At level one, pain is usually relieved with NSAIDs or paracetamol, which can be effective for minor pain such as headaches, muscle aches, or minor injuries. If the pain does not respond to these drugs, we can move to step two where milder opioids such as tramadol are given, which can reduce the pain without causing many harmful side effects. At the third level, once the pain becomes more severe, stronger opioids such as morphine, oxycodone, or fentanyl may be required, which can be very effective in reducing pain, for example in patients with tumors or severe injuries. The advantage of a stepwise approach is that it allows for the gradual and effective use of analgesics while minimizing overuse and side effects.

**Figure 6:**
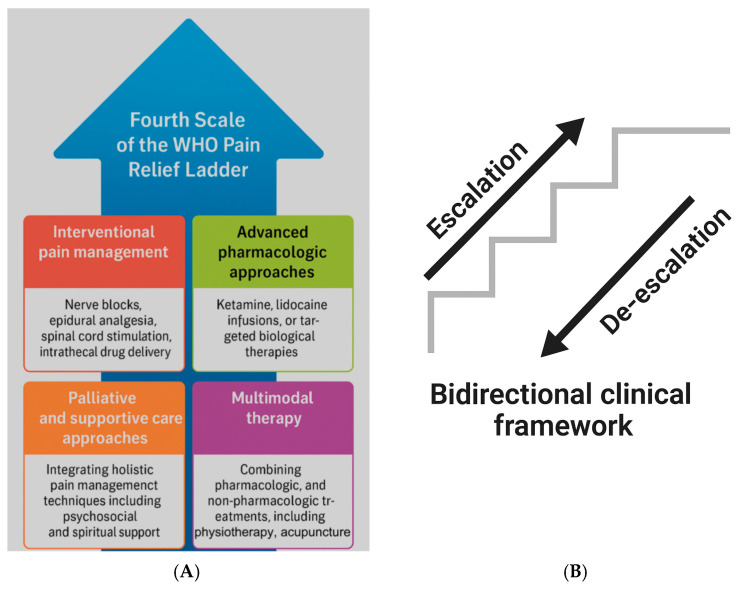
(**A**) The fourth step with interventional strategies. The fourth step represents the interventional and advanced strategies for refractory pain, including surgical procedures and neuromodulation; (**B**) the ladder also reflects a bidirectional approach that allows de-escalation when pain improves [50].

While the original World Health Organization (WHO) ladder consisted of three steps, the revised version is supplemented with a fourth scale. This includes the invasive procedures for treating severe pain (Figure 6A). Part B in the figure refers to the important fact that the level of analgesia can be reduced (de-escalation) if the clinical condition improves [50] (Figure 6B).

Surgical intervention or minimally invasive procedures could provide further pain relief if the applied medication does not work well [51].

The original WHO ladder was unidirectional, starting from the lowest step with NSAIDs including COX inhibitors and acetaminophen, and heading towards the strong opioids, depending on the patient’s pain. The updated WHO analgesic ladder focuses on the quality of life and is intended as a bidirectional approach; clinicians should also instigate a de-escalation of the treatment in cases of chronic pain resolution.

The use of a combination of medicines is an important element in the treatment of pain. A combination of medicines used for pain relief can help to reduce pain more quickly and effectively when a single medicine is not enough. Combining opioids and non-opioids, for example NSAIDs with a weaker opioid such as tramadol, can often relieve pain while reducing the doses and side effects of each drug. For chronic pain, the ad hoc use of analgesics is discouraged because it does not provide continuous and adequate pain relief. Instead of an ad hoc approach, it is important that pain relief is planned and controlled so that the patient does not suffer unnecessarily.

The effectiveness of the treatment also depends on the way in which the painkillers are used. Different routes of administration ensure that the drugs work as quickly and for as long as possible. Rectal administration, for example in the form of suppositories, is particularly useful if the patient is unable to take medication orally, for example because of vomiting or difficulty swallowing. Suppositories are rapidly absorbed through the intestinal wall, providing a rapid analgesic effect. The disadvantage is that not all patients are willing to accept suppositories, and not all painkillers are available in this form.

Oral drops are absorbed quickly and facilitate flexible dosing. This can be particularly useful when the pain is sudden and immediate relief is needed. Transdermal (through the skin) application using patches is one of the most convenient routes of administration. A well-accepted example is the fentanyl patch, which provides continuous pain relief for up to 72 h. This has the advantage of being convenient while not requiring frequent administration of the medication, but with the disadvantages that the initial effect is slower and the patches are not always comfortable for patients.

Long-acting medicines, such as long-acting opioids or fentanyl patches, can be of significant benefit in pain management, especially for people with persistent pain such as cancer patients. They have the advantage of being more convenient as they do not need to be administered frequently. Sustained-release preparations provide continuous pain relief which can help to reduce anxiety and worry for patients, as they can be assured that the pain relief will continue. Such preparations can help reduce the development of breakthrough pain (the sudden onset of pain). If breakthrough pain does occur, fast-acting opioids may be needed.

It is also important to consider the side effects of painkillers, especially in older people. Prolonged use of opioids can cause constipation, nausea, vomiting, drowsiness, respiratory depression, and an altered mental status, especially at higher doses. The main side effects of NSAIDs include damage to the stomach lining, high blood pressure, and kidney damage.

Pregabalin, gabapentin, and carbamazepine are anticonvulsants that are used to relieve neuropathic neuralgiform pain. They have an opiate-like spectrum of side effects (vomiting, constipation, respiratory depression, drowsiness) and may cause memory loss, difficulty concentrating, other cognitive problems (slowing of thinking), and depression [52]. Carbamazepine may also cause agranulocytosis, thrombocytopenia, and hyponatremia [53].

### Polypharmacy in the Management of Chronic Pain in Older Adults

Polypharmacy—commonly defined as the regular use of five or more medications—is highly prevalent among elderly patients, with estimates suggesting that 30–50% of individuals over the age of 65 meet this criterion [54,55]. In the context of chronic pain, polypharmacy poses challenges due to the need for the concurrent treatment of pain and multiple comorbid conditions such as cardiovascular disease, diabetes, or cognitive decline.

The use of multiple drugs increases the risk of adverse effects, including drug–drug interactions, therapeutic duplications, and cumulative organ toxicity. This is especially concerning when opioids are co-administered with other sedatives such as benzodiazepines, which can lead to respiratory depression, dizziness, and an increased fall risk. Furthermore, age-related renal and hepatic decline reduces drug clearance, thereby heightening the risk of drug accumulation and toxicity.

Despite these risks, a complete reduction in the medication burden is often not feasible in multimorbid geriatric patients. In such cases, the focus should shift from minimizing the drug count to rational prescribing, identifying high-risk combinations, and performing regular medication reviews. Pain medications should be selected and adjusted based not only on efficacy, but also on their safety profiles and interaction potential within the context of the patient’s full medication regimen.

Effective polypharmacy management includes periodic medication reconciliation, deprescribing inappropriate drugs, optimizing dosages, and prioritizing non-pharmacological interventions when feasible. Interprofessional collaboration—especially involving nurses, pharmacists, and geriatricians—is essential to ensure that the analgesic regimens are both effective and safe in this vulnerable population.

Regular assessment of the treatment efficacy, side effects, and drug interactions is critical in minimizing harm and ensuring the success of pain management strategies in elderly patients.

## 14. The Difficulties of Pain Relief from the Perspective of Patients and Clinicians

Chronic pain management presents many challenges for both patients and clinicians. Many aspects of long-term pain management can cause problems that affect the effectiveness of the treatment and the patient’s quality of life. One of the biggest difficulties for patients is accepting the sensation of pain and communicating it accurately to their doctors. Chronic pain is often a subjective phenomenon, and patients are not always able to accurately describe its nature, intensity, or impact on other aspects of their lives, which can reduce the effectiveness of pain management [56]. In addition, many patients may be frustrated if the pain relief does not result in an immediate or lasting improvement, as the effects of the painkillers are not always predictable and long-term adherence to treatment requires patience.

Side effects can also be a common problem for patients. The long-term use of painkillers often leads to dizziness, drowsiness, indigestion, or reduced social and work activities, which can significantly affect a patient’s quality of life. In addition, the long-term use of opioids and other stronger painkillers for chronic pain can cause patients to worry about developing an addiction, which can lead to further inhibition, fear, or rejection, even if the doctor believes that the treatment is safe. Patients often refuse to take painkillers properly if they feel they are not effective or if they experience too many side effects. Refusing to take painkillers for long periods can therefore lead to a non-adherence to the medical recommendations [57].

Management of chronic pain is a complex and time-consuming task for healthcare professionals. Pain management requires not only drug therapy but also a complex approach that may include physiotherapy, psychological support, and lifestyle changes. As well as the physical pain, psychological and social difficulties can also make diagnosis and treatment difficult. The use of painkillers is often not a complete solution, and doctors also need to consider individual factors to develop a tailored treatment plan.

In addition, doctors often do not have enough time or resources to deal with the complex issues involved in pain management. In addition to the medication, it is often necessary to involve several specialists, such as a pain specialist, psychologist, or physiotherapist, but these integrated treatments are not always available within the healthcare system. Doctors must therefore consider the safety of the medication, the side effects, and the level of pain relief experienced by the patient, as well as the risks associated with the long-term use of the medication, such as the development of tolerance or drug interactions. It is a common experience that physicians are reluctant to give patients adequate analgesia, despite their primary duty to reduce patient suffering [58].

One of the ethical and legal aspects of chronic pain management is the use of opioids, which carries the risk of addiction. Doctors need to monitor patients’ conditions to avoid overuse and to ensure that the treatment does not lead to situations that could have legal consequences. Another concern is that the use of opioids can lead to a loss of patient autonomy. Maintaining doctor–patient trust and ensuring that the appropriate medication is given is therefore key for both parties. A continuous review of the treatment and the use of alternative pain management options can help to ensure that pain relief remains effective and safe.

### 14.1. Limitations of Existing Guidelines in Geriatric Pain Management

While numerous clinical guidelines have been developed for the management of chronic pain, their consistent implementation in geriatric care remains limited. One emerging observation from clinical practice is the discrepancy between the evidence-based recommendations and their real-world applicability in frail older patients, particularly in those with multimorbidity, cognitive impairment, or polypharmacy. Standard protocols often fail to accommodate the unique physiological and psychosocial contexts of elderly care, such as altered pharmacokinetics, functional decline, or communication barriers.

This review underscores the need to adapt guideline-based approaches to the nuanced realities of geriatric settings. The development of personalized, context-sensitive strategies—grounded in interdisciplinary collaboration and continuous assessment—is essential in improving the outcomes for older adults with chronic pain. The current lack of emphasis on individualized care within many national and international guidelines represents a significant gap that warrants further research and clinical attention.

### 14.2. Clinical Challenges and Ethical Reflections in Elderly Pain Management

In the context of elderly pain management, ‘ethical reflections’ refer to the delicate balance between respecting the patient’s wishes—such as concerns about confusion or sedation from strong analgesics—and the clinician’s responsibility to provide effective pain relief. These reflections involve negotiating shared decisions where patient autonomy, clinical judgment, and potential side effects must be carefully weighed.

To provide a more comprehensive contextual analysis of chronic pain management in elderly individuals, a concise case scenario is hereby presented. This scenario aims to illustrate the diagnostic and therapeutic challenges that are particularly prevalent in frail or cognitively impaired patients.

Case study:

Mrs. K., an 84-year-old woman afflicted with severe osteoarthritis, moderate vascular dementia, and chronic kidney disease, is a resident in a long-term care facility. The subject is predominantly non-verbal, yet exhibits indications of distress, including facial grimacing, reluctance to be mobilized, and disrupted sleep patterns. Notwithstanding the manifest behavioral indications, the patient’s pain remained inadequately managed for a period of several weeks. The nurse initiated the PAINAD scoring, which indicated moderate pain. The utilization of non-opioid analgesics proved ineffective or was contraindicated due to renal complications. Following a multidisciplinary consultation, the patient was administered a low-dose transdermal opioid patch in combination with physiotherapy and psychological support. This combination therapy resulted in a significant improvement in the patient’s mobility and mood.

Discussion:

This case demonstrates the diagnostic uncertainty and therapeutic limitations that are frequently encountered in elderly patients suffering from cognitive decline. It is imperative that both creativity and caution are employed in the management of pain, given the existence of communication barriers, comorbidities, and age-related pharmacokinetic changes.

Moreover, ethical considerations arise in relation to the balancing of the imperative to alleviate suffering with the risk of overmedication or adverse effects. The question that arises is how to ensure informed consent in circumstances where cognitive impairment limits autonomy. These dilemmas demand clinical judgement as well as institutional support for individualized, interdisciplinary care planning.

The integration of case-based education into clinicians’ training, coupled with the promotion of reflective practice, has been demonstrated to engender a more profound comprehension of the distinctive intricacies inherent in geriatric pain management. Such efforts may ultimately engender improvements in both the outcomes and the compassion in care delivery.

### 14.3. Barriers to Effective Chronic Pain Management in the Elderly

Despite a growing awareness of chronic pain as a critical health issue in older adults, numerous barriers continue to impede effective assessment and treatment. These obstacles arise at the patient, provider, and healthcare system levels and must be recognized and addressed to optimize outcomes.

Patient-related barriers include the frequent underreporting of pain, often due to the belief that pain is a natural consequence of aging or the fear of being a burden. Communication deficits, especially in individuals with dementia or cognitive impairment, further complicate accurate symptom reporting. Fear of opioid dependence, concerns about side effects, and a limited understanding of the treatment options may also reduce treatment adherence.

Clinician-related barriers involve the under-recognition or misattribution of pain symptoms, therapeutic inertia, and insufficient training in geriatric pain assessment. Physicians may hesitate to prescribe effective analgesics due to concerns about polypharmacy, drug interactions, or regulatory scrutiny—particularly in the case of opioids. Time constraints in clinical settings also limit the opportunity for comprehensive pain evaluation and individualized care planning [59].

Nurses, who are often on the front line of pain assessments, may also encounter institutional, educational, and resource-related barriers to implementing evidence-based practices in pain management [60].

System-level barriers encompass fragmented healthcare services, the insufficient integration of multidisciplinary pain management teams, and limited access to non-pharmacological therapies such as physiotherapy, psychological support, or occupational therapy. Financial constraints and under-resourced long-term care facilities may also hinder appropriate and sustained pain control.

Together, these barriers contribute to the undertreatment of chronic pain in older adults, exacerbating physical disability, psychological distress, and social withdrawal. Addressing these challenges requires a multifaceted approach that includes professional education, structural reforms, improved communication strategies, and the active involvement of caregivers and family members in care planning.

## 15. Non-Pharmacological Approaches to Pain Management

Although medication offers an effective solution to pain management, a growing body of research and clinical experience suggests that non-pharmacological therapies can also play an essential role in pain management, particularly in the management of chronic pain among elderly patients where pharmacologic options are often limited due to side effects or polypharmacy concerns [61].

Physiotherapy is one of the most widely used non-pharmacological treatments. Various types of massage, heat and cold treatments, and physiotherapy exercises can provide significant pain relief, especially for musculoskeletal pain such as arthritis or back pain [62]. Electrotherapy such as transcutaneous electrical nerve stimulation (TENS) can also be successfully used to reduce pain [63].

Cognitive behavioral therapy (CBT) is a psychological treatment that can help to manage negative psychological responses to pain. CBT aims to teach people to live more positively with pain by changing their thoughts and behaviors around pain. These techniques can help to reduce the perception of pain and reduce pain-related anxiety and stress [64].

Acupuncture, a well-known method of traditional Chinese medicine, reduces pain by inserting needles into specific points on the body. Although the mechanism of acupuncture is not fully understood, a large body of research and clinical practice suggests that it can be effective in relieving various types of pain, such as chronic back pain, migraines, or joint pain [65]. Relaxation techniques such as deep breathing, meditation, and visualization can also help to reduce pain [66,67].

**Figure 7:**
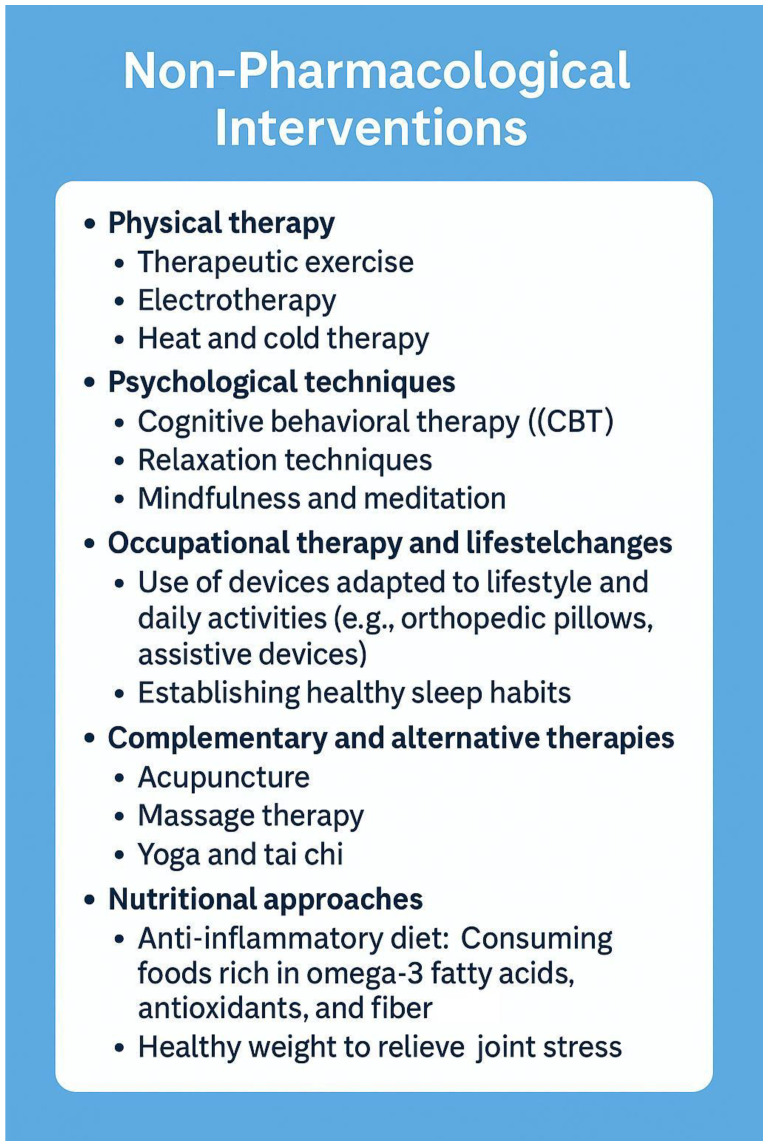
Non-pharmacological pain management (own illustration) [69].

Biofeedback technology allows patients to consciously control their own physiological functions, such as pulse, muscle tension, or breathing. The control of these functions can help in pain management as the patient can learn to control their pain responses [68] (Figure 7).

## 16. Summary of Evidence and Recommendations

To support clinical decision-making in the management of chronic pain in older adults, we have compiled a summary of the interventions discussed in this review, along with their corresponding level of evidence and strength of recommendation.

The grading is based on the available high-quality sources, including systematic reviews, meta-analyses, and guidelines from organizations such as the World Health Organization (WHO), the American Geriatrics Society (AGS), the National Institute for Health and Care Excellence (NICE), and Cochrane reviews.

Pharmacological and non-pharmacological interventions have been evaluated with respect to their efficacy, safety, and relevance in the geriatric population. The table below provides a practical reference for clinicians to assess the current quality of the evidence and guide individualized treatment planning (Table 3).

## 17. Future Directions in Geriatric Pain Management

Advancing pain management in the elderly population requires clinical, ethical, and health policy considerations alike. A central challenge lies in balancing effective pain relief with the minimization of side effects, while also better incorporating the preferences and lived experiences of older adults.

Future research should place a stronger emphasis on the non-pharmacological pain management strategies, such as physiotherapy, psychological interventions (e.g., cognitive behavioral therapy), and integrative approaches like acupuncture, relaxation techniques, or music therapy. These modalities are often better tolerated by older patients and may be used alone or in combination with pharmacological treatments. However, the current evidence base remains limited or of low quality in many cases, underlining the need for well-designed studies to evaluate the efficacy and safety of these alternative approaches specifically in geriatric populations.

Moreover, future clinical trials should not focus solely on numerical reductions in pain intensity. Instead, outcomes that truly matter to older patients should be prioritized-including improvements in their quality of life, the preservation of functional independence, psychosocial well-being, and the ability to maintain everyday activities. These patient-centered outcomes will enable more meaningful and individualized decision-making in pain care.

Finally, involving older patients in therapeutic decisions must go beyond a formal requirement and become an integral part of routine clinical practice. Achieving this goal will require education, practical guidelines, and interdisciplinary collaboration, all of which are essential to support a more personalized, humane, and effective approach to pain management in later life.

## 18. Conclusions

Chronic pain is highly prevalent among older adults and presents unique diagnostic and therapeutic challenges. Its assessment requires a multidimensional approach, as the perception and expression of pain in elderly patients are often shaped by cognitive decline, depression, and social isolation. Importantly, pain is always as intense as the patient perceives it to be.

Effective pain management in geriatric patients must rely on a balanced combination of pharmacological and non-pharmacological interventions. Pharmacological options include minor and major analgesics, as well as adjuvant agents. However, age-related physiological changes-particularly renal impairment-and the high prevalence of polypharmacy significantly increase the risk of adverse effects. Therefore, treatment must be carefully tailored, monitored, and adjusted to each patient’s functional status, comorbidities, and personal circumstances.

While pharmacological agents often provide rapid relief, non-drug methods such as physiotherapy, cognitive behavioral therapy, and psychosocial support serve as important adjuncts. The most successful outcomes are achieved through individualized, multimodal strategies that address the biological, psychological, and social aspects of aging.

Ultimately, effective chronic pain management in the elderly is not solely about symptom control, but about improving quality of life, preserving autonomy, and addressing the complex realities of aging with empathy and clinical precision.

## Figures and Tables

**Table 1:** NSAID analgesics commonly used in elderly patients.

Active Ingredient	Recommended Dosages	Main Indications	Common Side Effects
Ibuprofen	200–400 mg every 4–6 h max. 3200 mg/day	Mild to moderate pain, fever, inflammatory conditions	Gastrointestinal discomfort, dyspepsia, ulcers
Naproxen	250–500 mg every 12 h max. 1500 mg/day	Arthritis (OA, RA), dysmenorrhea, acute pain	Gastritis, dizziness, kidney problems
Diclofenac	50 mg 2–3 times/day max. 150 mg/day	Arthritis, postoperative pain, muscle pain	Liver enzyme elevation, gastric issues, hypertension
Meloxicam	7.5–15 mg/day	Osteoarthritis, rheumatoid arthritis	Gastrointestinal bleeding, dizziness, hypertension
Celecoxib	100–200 mg 1–2 times/day	Osteoarthritis, rheumatoid arthritis, acute pain	Reduced GI side effects, cardiovascular risks
Aspirin	325–650 mg every 4–6 h max. 4 g/day	Fever, mild pain, platelet aggregation inhibition	Gastritis, tinnitus, Reye’s syndrome in children

Note: Dosage adjustments may be necessary in older adults due to altered renal function and increased gastrointestinal sensitivity. Abbreviations: OA = osteoarthritis; RA = rheumatoid arthritis; GI = gastrointestinal. The dosages, indications, and side effects listed in Table 1 are based on commonly accepted prescribing information and guidelines from the American Geriatrics Society, Beers Criteria, and drug monographs such as Lexicomp^®^ (Hudson, OH, USA) and Micromedex^®^ (Greenwood Village, CO, USA).

**Table 2:** Weak and strong opioid analgesics. The doses indicated are for adults and may vary according to individual tolerance, pain level, and method of administration.

Active Ingredient	Category	Recommended Dosages	Routes of Administration	Indications	Common Side Effects
Tramadol	Weak opioid	50–100 mg every 4–6 h (max. 400 mg/day)	Oral, intravenous, intramuscular	Moderate to moderately severe pain	Nausea, dizziness, constipation
Codeine	Weak opioid	15–60 mg every 4–6 h (max. 240 mg/day)	Oral	Cough suppression, mild to moderate pain	Constipation, drowsiness, nausea
Tapentadol	Weak-to-strong opioid	50–100 mg every 4–6 h (max. 500 mg/day)	Oral	Moderate to severe pain, neuropathic pain	Dizziness, nausea, constipation
Morphine	Strong opioid	5–10 mg every 4 h (oral), 2–4 mg every 1–2 h (IV)	Oral, intravenous, subcutaneous, intramuscular	Severe acute or chronic pain, palliative care	Respiratory depression, constipation, sedation
Oxycodone	Strong opioid	5–10 mg every 4–6 h (max. 80 mg/day)	Oral, intravenous	Severe pain, e.g., postoperative or cancer pain	Respiratory depression, dizziness, nausea
Fentanyl	Strong opioid	25–100 µg/hour (transdermal patch), 50–100 µg IV	Transdermal, intravenous, intranasal, sublingual	Chronic or breakthrough pain	Respiratory depression, muscle rigidity, constipation
Buprenorphine	Moderate opioid	5–20 µg/hour (transdermal), 0.2–0.4 mg (sublingual)	Transdermal, sublingual	Moderate to severe pain, opioid dependence	Dizziness, nausea, constipation
Hydromorphone	Strong opioid	2–4 mg every 4–6 h (oral), 0.2–1 mg IV	Oral, intravenous	Severe pain, cancer-related pain	Respiratory depression, drowsiness, nausea
Methadone	Strong opioid	2.5–10 mg every 8–12 h (oral)	Oral, intravenous	Chronic pain, opioid dependence treatment	QT prolongation, respiratory depression, constipation
Meperidine	Strong opioid	50–150 mg every 3–4 h (max. 600 mg/day)	Oral, intravenous	Severe acute pain (limited use)	Tremors, seizures, respiratory depression

Note: Opioids are grouped for clarity as weak or strong. Dosing should always consider renal function, hepatic impairment, and patient frailty. Abbreviations: IV = intravenous; OA = osteoarthritis; RA = rheumatoid arthritis; SR = sustained-release; IR = immediate-release. (Compiled from multiple sources, including the WHO analgesic ladder, American Geriatrics Society guidelines, and drug monographs in Lexicomp^®^ (Hudson, OH, USA), Micromedex^®^ (Greenwood Village, CO, USA), and UpToDate^®^ (Waltham, MA, USA)).

**Table 3:** Summary of evidence and recommendations (compiled from GRADE [Grading of Recommendations Assessment, Development and Evaluation] and Oxford CEBM [Centre for Evidence-Based Medicine] guidelines).

Intervention	Type	Level of Evidence	Strength of Recommendation
Paracetamol	Pharmacological	Moderate (Level B)	Conditional
NSAIDs (e.g., ibuprofen, diclofenac)	Pharmacological	High (Level A)	Strong (with caution)
Opioids (e.g., morphine, tramadol)	Pharmacological	High (Level A)	Strong (short-term use)
Antidepressants (e.g., amitriptyline)	Adjuvant	Moderate (Level B)	Conditional
Anticonvulsants (e.g., gabapentin)	Adjuvant	Moderate (Level B)	Conditional
Physiotherapy	Non-pharmacological	High (Level A)	Strong
Cognitive Behavioral Therapy (CBT)	Non-pharmacological	High (Level A)	Strong
Acupuncture	Non-pharmacological	Low–Moderate (B–C)	Conditional
Transcutaneous Electrical Nerve Stimulation (TENS)	Non-pharmacological	Moderate (Level B)	Conditional

Note: Levels of evidence are based on standardized grading systems such as GRADE and the Oxford Centre for Evidence-Based Medicine [70,71].
